# Domain-specific regulation of *foxP2 *CNS expression by *lef1*

**DOI:** 10.1186/1471-213X-8-103

**Published:** 2008-10-24

**Authors:** Joshua L Bonkowsky, Xu Wang, Esther Fujimoto, Ji Eun Lee, Chi-Bin Chien, Richard I Dorsky

**Affiliations:** 1 Division of Pediatric Neurology, Department of Pediatrics, University of Utah School of Medicine, Salt Lake City, Utah 84108, USA; 2 Department of Neurobiology and Anatomy, University of Utah School of Medicine, Salt Lake City, Utah 84132, USA; 3 Neurogenetics Laboratory, Department of Neurosciences, University of California, San Diego, La Jolla, California 92093, USA

## Abstract

**Background:**

*FOXP2 *is a forkhead transcription factor critical for normal development of language in humans, but little is known of its broader function and regulation during central nervous system (CNS) development. We report here that *lef1*, a member of the Lef/Tcf family of transcription factors activated by Wnt signaling, regulates *foxP2 *during embryogenesis, and we isolate novel *foxP2 *enhancers which are *lef1*-dependent.

**Results:**

Loss, knock down, or inhibition of *lef1 *led to loss of *foxP2 *expression. We isolated DNA fragments from the *foxP2 *genomic region that function as enhancers to drive GFP expression in the CNS during development, including in the telencephalon, diencephalon, eye, tectum, and hindbrain. Three of these enhancers, foxP2-enhancerA.1, foxP2-enhancerB, and foxP2-enhancerD, contain putative Lef1 binding sites, and are regulated by *lef1*. However, two other genomic fragments containing Lef1 sites failed to function *in vivo *as enhancers. Chromatin immunoprecipitation confirmed that Lef1 binds to sites in foxP2-enhancerA.1 and foxP2-enhancerB.

**Conclusion:**

This work shows that *lef1 *is necessary for expression of *foxP2 *in the tectum, mid-hindbrain boundary, and hindbrain during CNS development, and is the first insight into the upstream regulation of *foxP2 *during development. We also demonstrate that *in silico *prediction of potential *lef1 *binding sites poorly predicts their ability to function *in vivo *as enhancers. The *foxP2 *enhancers we identified will allow dissection of *foxP2*'s role during CNS development.

## Background

*FOXP2 *is a forkhead domain transcription factor whose mutation has been associated with severe deficits in language [1-6]. Its cloning and expression during development have been described in humans, mouse, songbird, frog, medaka, and zebrafish [7-16]. CNS expression during development in the different vertebrate species is remarkably similar, with conserved expression in the telencephalon, basal ganglia, thalamus, tectum, tegmentum, cerebellum, and hindbrain. However, the function of *FOXP2 *during CNS development is poorly understood. Homozygous knockout or point mutation of Foxp2 in mice leads to early postnatal death, with reports of disordered Purkinje cell layers [17-19] and smaller cerebellar size [18,20]. In songbirds, *FoxP2 *appears to be necessary for vocal learning and is expressed in neurons during active song learning [9,21,22].

The neural circuits and genetic cascades in which *FOXP2 *participates remain uncharacterized. Chromatin immunoprecipitation methods have identified potential downstream targets of *FOXP2 *[23,24], but *in vivo *function and importance of the identified targets is uncertain. To understand in greater detail the role of *foxP2 *in CNS development, we sought to identify how *foxP2 *expression is regulated. The conservation of six predicted *lef1 *binding sites between pufferfish, zebrafish, mouse, and human in the *foxP2 *genomic region (this study; [25], and the overlapping expression of *lef1 *and *foxP2 *in the zebrafish CNS during development, led us to consider whether *lef1 *might regulate *foxP2*. Lef1 is a transcription factor activated by the canonical Wnt/β-catenin signaling pathway, which has been shown to play a critical role in proliferation, tissue patterning, CNS neuronal cell fate specification, and axon pathfinding [26]. We found that loss or knockdown of *lef1 *led to a loss of *foxP2 *expression in the tectum, mid-hindbrain boundary, and hindbrain. Of six conserved potential *lef1 *binding sites predicted in the *foxP2 *genomic region [25], we show that only three lie in genomic fragments that function *in vivo *as enhancers, underscoring the importance of *in vivo *testing of predicted enhancers. Using chromatin immunoprecipitation (ChIP), we demonstrated that *lef1 *can bind directly to the functional enhancer sites, and showed that in the absence of *lef1 *these enhancers fail to function. The *foxP2 *enhancers will be useful for dissection of *foxP2 *function by allowing detailed analysis of axon pathfinding and synaptogenesis in *foxP2*-expressing neurons.

## Results

### *foxP2 *and *lef1 *have sequential and overlapping expression in the CNS during embryogenesis

**Figure 1:**
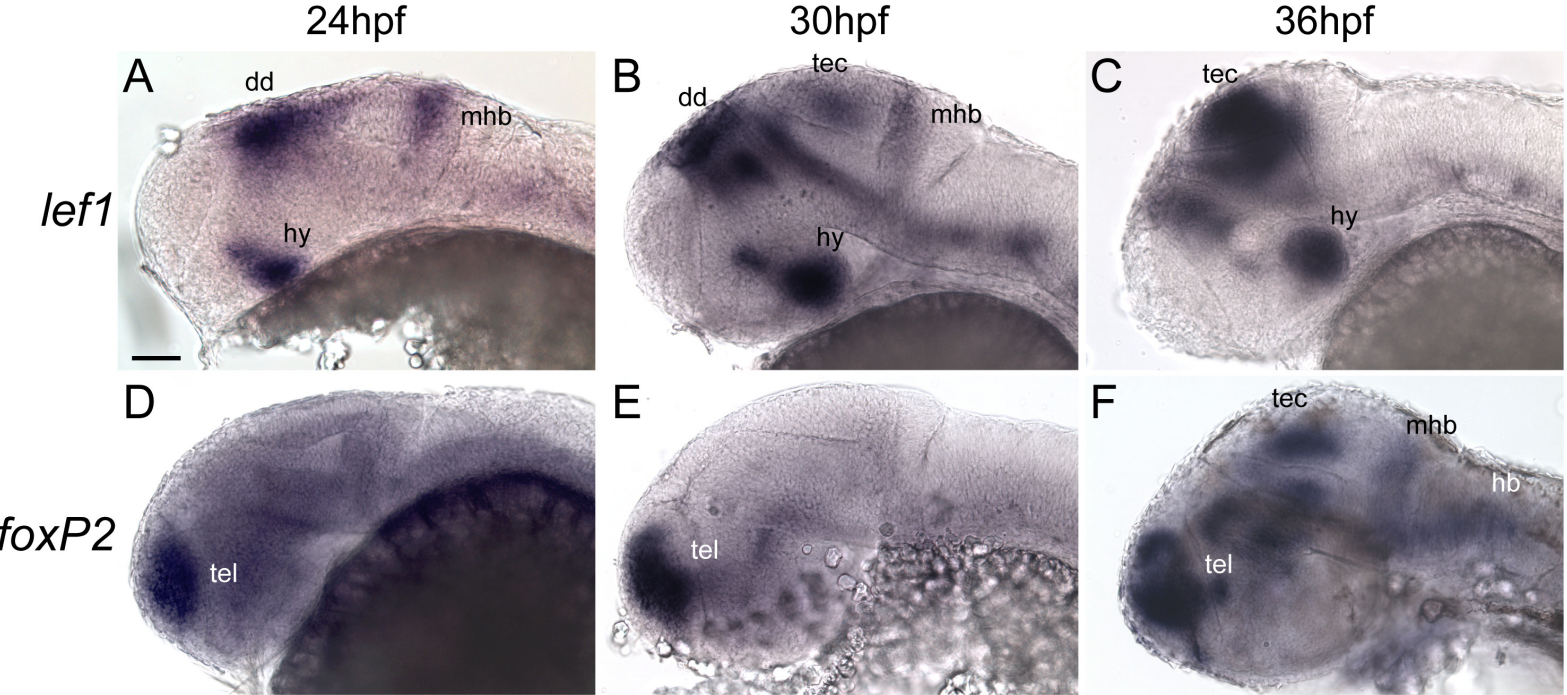
**Sequential expression of *foxP2 *and *lef1 *in the CNS during embryogenesis**. Whole-mount *in situs *for *lef1 *(A-C) and *foxP2 *(D-F) at 24 hpf, 30 hpf, and 36 hpf. Lateral views, anterior to left, dorsal up; eyes have been removed to facilitate visualization. Scale bar = 50 μm. (Abbreviations: dd, dorsal diencephalon; hb, hindbrain; hy, hypothalamus; mhb, mid-hindbrain boundary; tec, tectum; tel, telencephalon.) (A-C): *lef1 *is expressed in the hypothalamus, dorsal midbrain, and MHB at 24 hpf, with expression extending to the tectum at 30 hpf. By 36 hpf expression is confined primarily to the hypothalamus and tectum. (D-F): *foxP2 *is expressed in the tectum and MHB starting at 36 hpf. Earlier expression (24 hpf and 30 hpf) is confined to the telencephalon.

**Figure 2:**
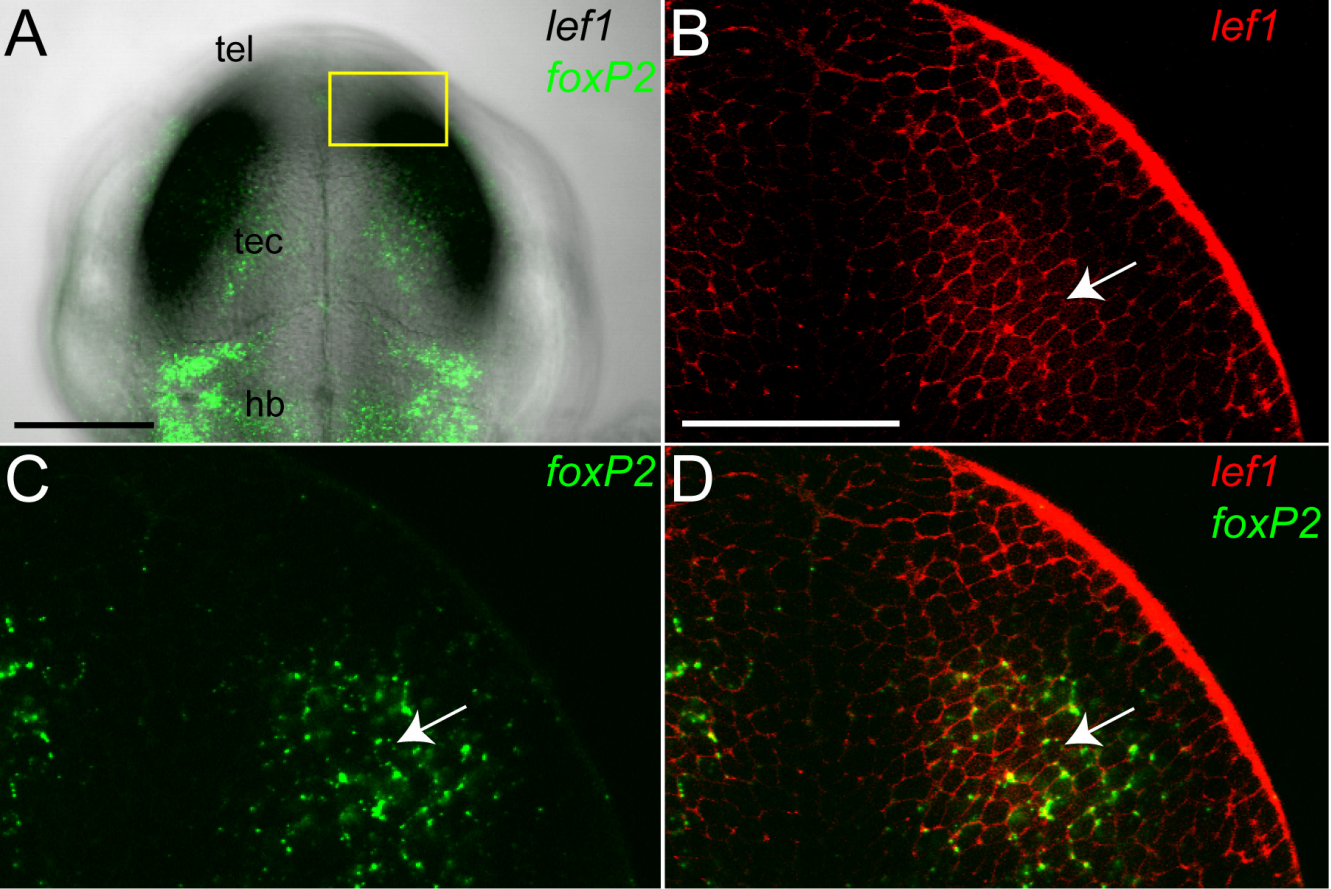
**Co-expression of *foxP2 *and *lef1 *in the tectum**. Whole-mount double *in situ *confocal imaging for *foxP2 *and *lef1 *at 36 hpf, dorsal views, anterior up. Abbreviations: hb, hindbrain; tec, tectum; tel, telencephalon. (A) Z-stack projection of *foxP2 *(green) overlaid on brightfield image of *lef1 *expression in the tectum. The region shown in higher magnification in (B-D) is boxed in yellow. Scale bar = 50 μm. (B-D) Single optical plane showing *lef1 *(red- BM Purple), *foxP2 *(green- Alexa 488), and co-expression in the tectum. Arrow points to a representative co-expressing cell. Scale bar = 25 μm.

We noted that 6 binding sites for Tcf/Lef transcription factors are conserved between mouse and pufferfish in the *FOXP2 *genomic region, which is more than for any other putative Tcf/Lef target gene (Table S6 of [25]. This finding, and our prior observations that *foxP2 *and *lef1 *are expressed in the tectum and hindbrain during CNS embryogenesis [7,27], raised the possibility that *lef1 *might regulate *foxP2 *expression. We performed *in situ *labeling for *foxP2 *and *lef1 *to investigate whether they shared temporal and/or spatial domains of expression. We found that *lef1 *is expressed in the mid-hindbrain boundary (MHB) starting at 24 hpf, and in the tectum starting at 30 hpf (Figure 1A–C). *foxP2 *expression in the MHB and tectum becomes apparent at 36 hpf (Figure 1D–F). Other domains of *lef1 *and *foxP2 *expression, including the telencephalon, hypothalamus, and dorsal diencephalon, had non-overlapping expression. To confirm that *foxP2 *and *lef1 *are indeed co-expressed in the same cells, rather than in distinct subsets of cells in the same region, we performed double *in situs*, and found that *foxP2 *and *lef1 *are co-expressed in tectal cells at 36 hpf (Figure 2A–D). This sequentially overlapping pattern of expression in the MHB, and contemporaneous overlap in the tectum, confirmed that *lef1 *was present in a relevant pattern to potentially direct *foxP2 *expression.

### Knockdown of *lef1 *causes loss of *foxP2 *expression

**Figure 3:**
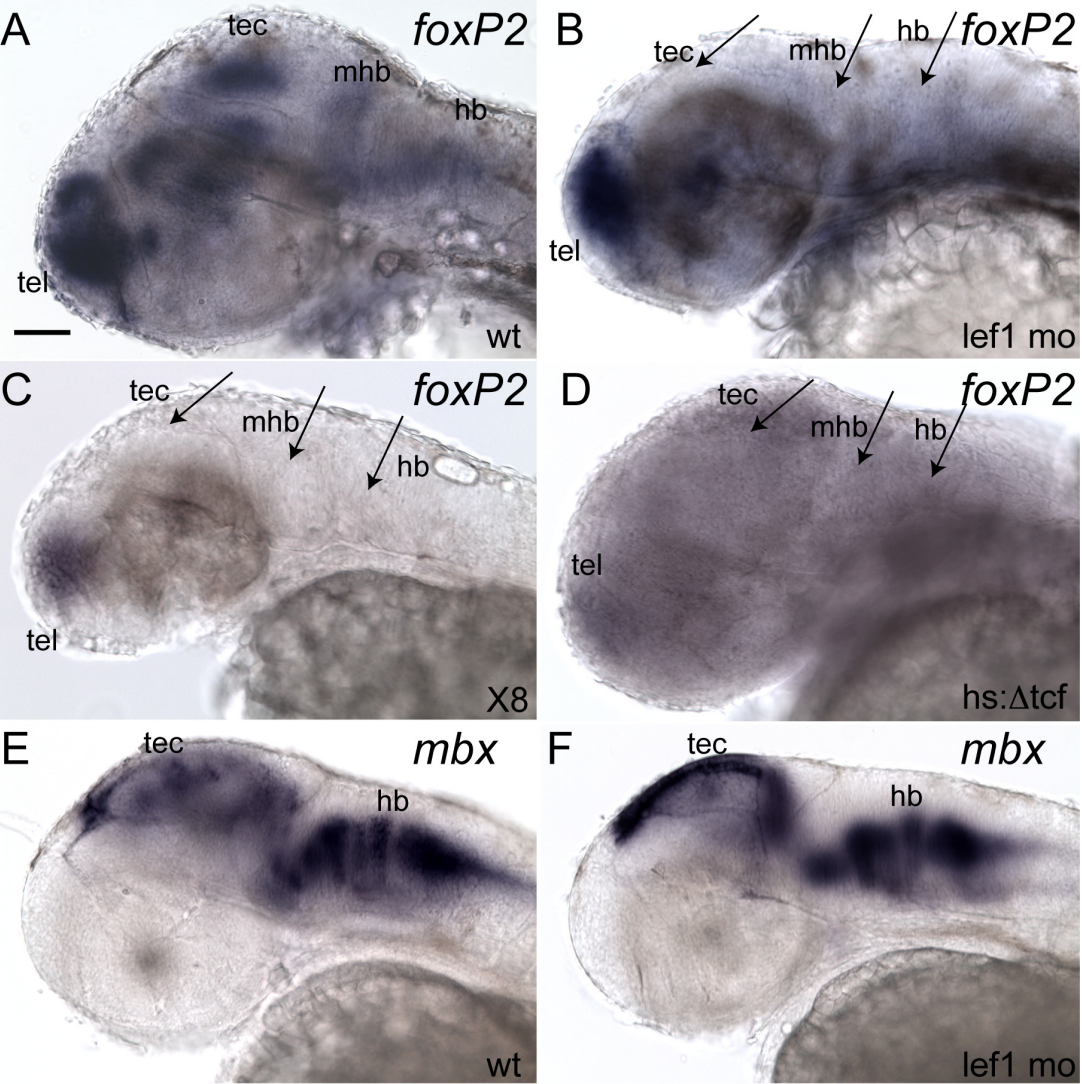
**Loss of *lef1 *leads to absent *foxP2 *expression in the tectum, mid-hindbrain boundary, and hindbrain**. Whole-mount *in situ*s at 36 hpf; anterior to left, dorsal up, eyes removed. Scale bar = 50 μm. (Conditions: hs:Δtcf, Tg*(hsp70l:Δtcf-GFP*)^w26^; *lef1 *mo, *lef1 *morphants; wt, wild type; x8, homozygous Df(*LG01*)^x8^. Abbreviations: hb, hindbrain; mhb, mid-hindbrain boundary; tec, tectum; tel, telencephalon.) (A, B) *lef1 *morphant (B) lacks expression in tectum, MHB, and hindbrain (*arrows*), compared to wild-type (A). (C) Df(*LG01*)^x8 ^homozygote lacks expression in tectum, MHB, and hindbrain (*arrows*). (D) Tg*(hsp70l:Δtcf:GFP*)^w26 ^embryo, three hours post-heat shock, lacks expression in tectum, MHB, and hindbrain (*arrows*). (E, F) *mbx in situs*; staining in tectum and hindbrain is indistinguishable between wild type and *lef1 *morphants (F).

Based on this overlap of expression, we hypothesized that *lef1 *might regulate *foxP2*. To test this, we used several approaches. First, we used a morpholino to knockdown *lef1 *[27], and evaluated *foxP2 in situ *expression. Knockdown of *lef1 *causes a near-complete loss of *foxP2 *expression in the tectum, MHB, and hindbrain at 36 hpf (Figure 3A, B) in 85% of injected embryos (n = 61), compared with 0% of uninjected embryos (n = 53). This effect is specific to *lef1*, since a morpholino against a different Lef/Tcf family member, *tcf3b *[28], which is also expressed in the tectum and hindbrain [28], showed no effect on *foxP2 *expression (n = 48, data not shown). The loss of *foxP2 *expression in the hindbrain, where *lef1 *expression is not detectable by *in situ *(Figure 1A–C), is presumably an indirect effect, perhaps via loss of an inductive signal from the MHB. Alternatively, this could be a direct effect, for example, if *lef1 *is expressed at very low levels in the hindbrain, or if some *lef1*-positive neurons migrate from the MHB to the hindbrain.

To confirm that loss of *foxP2 *expression was due to knockdown of *lef1 *and not a non-specific morpholino effect, we utilized two alternative means to remove *lef1 *expression. First, we examined expression of *foxP2 *in Df(*LG01*)^x8 ^mutant embryos. Df(*LG01*)^x8 ^is a deletion on chromosome 1 which contains *lef1 *(but not *foxP2*); homozygous deficiency mutants do not express *lef1 *[27,29]. We found that homozygous Df(*LG01*)^x8 ^mutants do not express *foxP2 *in the tectum, MHB, or hindbrain (Figure 3C) (100%, n = 25 embryos).

Second, we examined whether a dominant negative construct which inhibits *lef1 *function would cause a loss of *foxP2 *expression. We used embryos carrying the transgene *hsp70l:Δtcf-GFP*, which expresses an N-terminal deletion of Tcf3a fused to GFP. In the absence of its N-terminal DNA-binding domain, Tcf3a acts as a dominant repressor of Wnt-mediated transcription [30]. Following heat-shock at 32 hpf for 1 hour, embryos were collected 3 hours post-heat shock. There was loss of *foxP2 *expression in the tectum, MHB, and hindbrain (Figure 3D) in 61% of transgenic embryos (n = 38), and in 0% of non-transgenic siblings (n = 82). Decrease of *foxP2 *telencephalic expression is presumably due to a dominant effect of the transgene, since the decrease was not seen using the morpholino or Df(*LG01*)^x8^.

The loss of *foxP2 *expression in the tectum and hindbrain is not secondary to an absence of cells, as expression of the tectal marker *mbx *[31] is indistinguishable between wild type and *lef1 *morphants (Figure 3E, F). Other markers for the tectum (*emx2*), and hindbrain (*isl1, zash1a*) of *lef1 *morphants also appear as wild type patterns and levels [27]; J.E.L. and R.I.D., unpublished data). Furthermore, the loss of *foxP2 *expression persists at 48 hpf (Additional File 1). These results show that loss of *lef1 *specifically causes loss of *foxP2 *expression in the tectum, MHB, and hindbrain.

### Identification of *foxP2 *genomic enhancers and Lef1 binding sites

**Figure 4:**
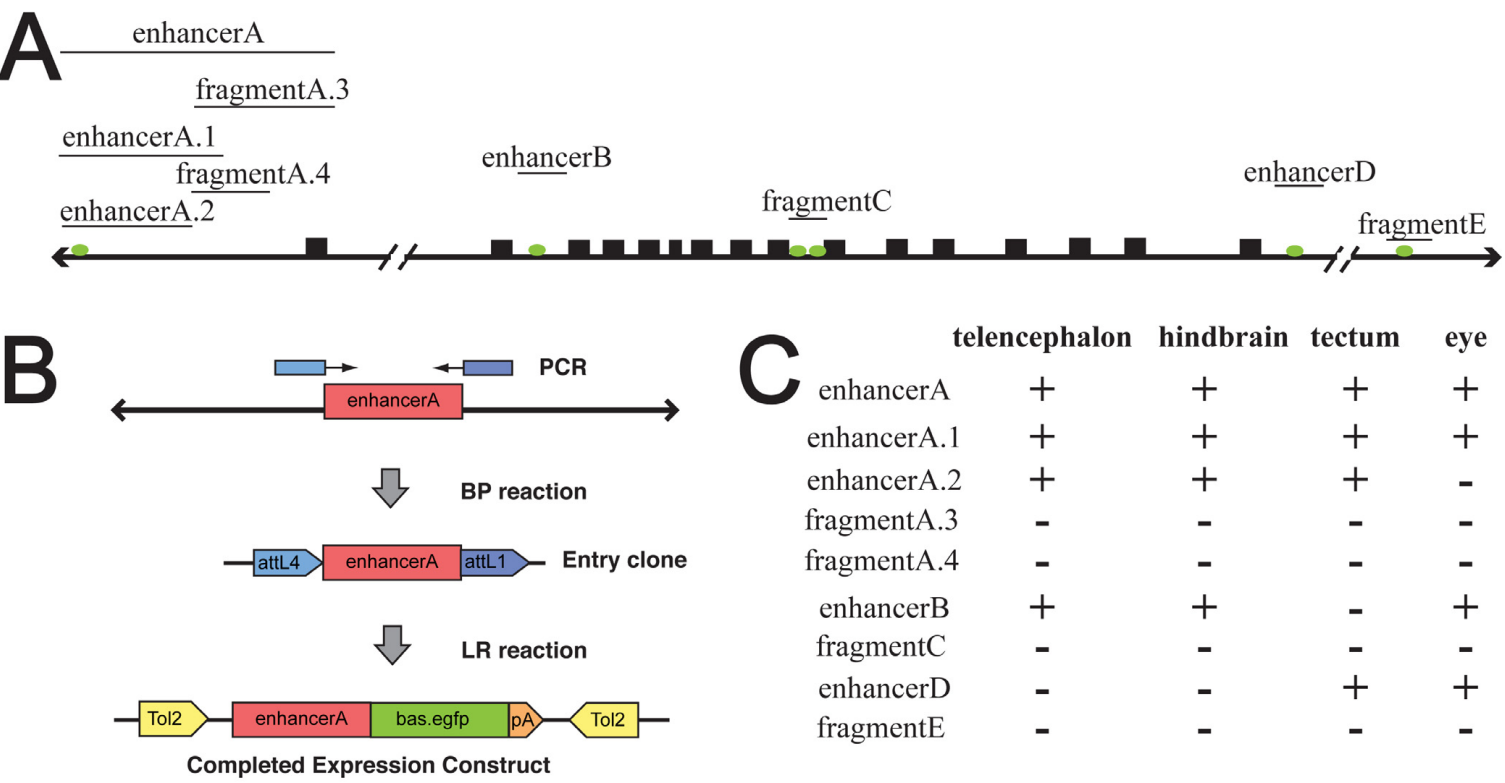
**Genomic structure and identification of enhancers of *foxP2***. (A) *foxP2 *genomic region (not to scale). Coding exons are shown as solid black boxes. Predicted *lef1 *binding sites are shown as ovals. DNA fragments tested for enhancer activity are shown. (B) Schematic of cloning strategy for DNA fragments. PCR primers for the genomic region of interest (red box) are designed with *attB4 *and *attB1 *sites (blue boxes), and the region is amplified and cloned using a BP reaction into an entry clone. A LR reaction is performed to recombine the entry clone (containing the genomic DNA fragment) into a Tol2 destination vector, placing the genomic fragment upstream of a basal promoter and EGFP (green box). (C) Summary table of expression patterns of the different enhancers at 72 hpf in the CNS.

6 potential conserved binding sites for *lef*/*tcf *family transcription factors were identified in the *FOXP2 *genomic locus of mouse and pufferfish using *in silico *analysis [25]. This algorithm (enhancer element locator) aligns transcription factor sequence sites from orthologous genomic regions between two species, with scoring of the sites determined by conservation, affinity, and clustering of sites. Based on conserved synteny between zebrafish, mouse, and pufferfish, we initially were able to identify 5 of these sites in the zebrafish *foxP2 *genomic locus using Sanger Centre genome assembly Zv6 (see Methods; Figure 4A).

Because the *foxP2 *genomic assembly is incomplete in the region upstream of the 2^nd ^coding exon (J.L.B., unpublished data), we sought to identify the sixth predicted *lef1 *binding site. In human *FOXP2 *this site is 8.8 kb upstream of the first coding exon. We obtained the sequence for this region in zebrafish from the unassembled BAC DKEY-116L11. Using the enhancer element locator algorithm [25], we compared the 9 kb regions immediately upstream of the first coding exon of *foxP2 *from the human and zebrafish genomes. We found conservation of this same element (zebrafish: ttgtgggctGCTTTCATCtgtgggttaa; human: atgatcagtGCTTTCATCtttattttaa) located 8.5 kb upstream of the first coding exon in zebrafish (contained within foxP2-enhancerA).

**Figure 5:**
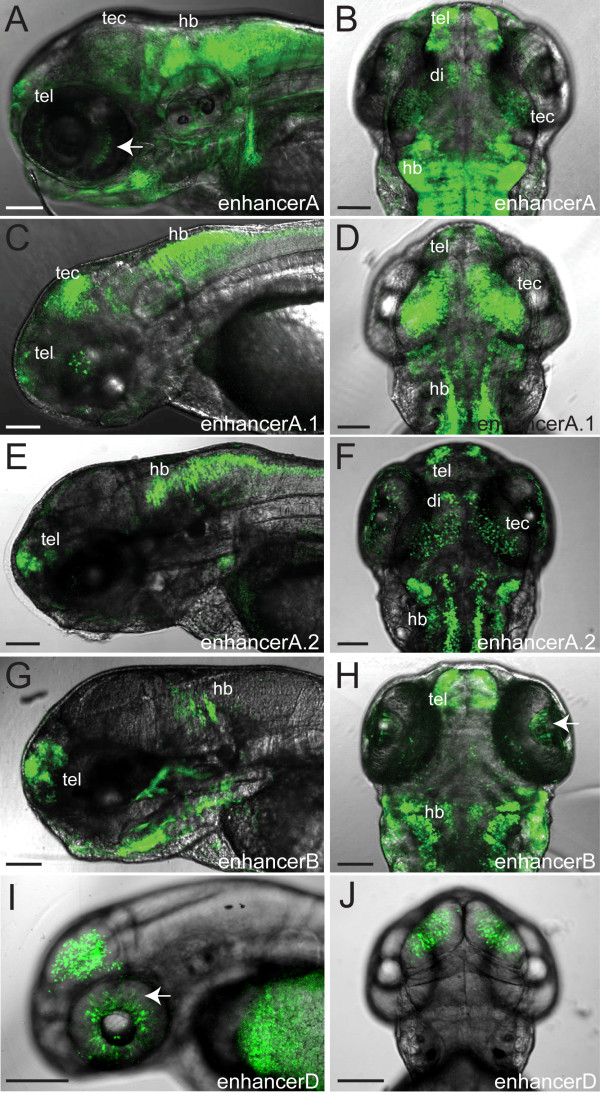
**Confocal live whole-mount images of *foxP2 *enhancers**. Pictures show GFP expression at 72 hpf (except foxP2-enhancerD, taken at 48 hpf). The eye has been removed in panels E and G to facilitate visualization. Scale bar = 100 μm. (A, C, E, G, I): lateral views, anterior left, dorsal up. (B, D, F, H, J): dorsal views, anterior up. (Abbreviations: di, diencephalon; hb, hindbrain; tec, tectum; tel, telencephalon; arrows, GFP-expressing cells in the eye. (A, B) Tg(*foxP2-enhancerA:EGFP)*^*zc42*^; (C, D) Tg(*foxP2-enhancerA.1:EGFP)*^*zc44*^; (E, F) Tg(*foxP2-enhancerA.2:EGFP*)^*zc46*^; (G, H) Tg(*foxP-enhancerB:EGFP)*^*zc41*^; (I, J) Tg*(foxP2-enhancerD:EGFP)*^*zc47*^.

To identify *foxP2 *enhancers, and to determine whether any of the potential *lef1 *sites might function *in vivo *in the context of enhancer elements, we cloned genomic fragments containing the sites into a Tol2 transposon-based vector (Figure 4B) [32-34]. To visualize expression controlled by the potential enhancers, each DNA fragment was cloned immediately upstream of GFP under control of a minimal promoter [32]. In total we cloned nine genomic DNA fragments (Figure 4A), from five different regions, containing the six predicted *lef1 *sites (two sites are contained in foxP2-fragmentC). To test for expression, we injected one cell stage embryos and assayed GFP expression from 12 hpf through 96 hpf (Figure 4C). Subsequent analysis used stable transgenic lines, which mostly reproduced the same patterns of expression. Three of the regions, foxP2-enhancerA, B, and D, drove GFP expression in patterns partially recapitulating *foxP2 *expression (Figure 5). Potential enhancers foxP2-fragmentC and foxP2-fragmentE had no expression.

foxP2-enhancerA drove expression in the eye, diencephalon, tectum, hindbrain, and telencephalon (Figure 5A, B). GFP expression was first noted in the telencephalon at 24 hpf, then at 48 hpf in the eye, tectum and hindbrain, becoming maximal at 72 hpf. By 80 hpf expression in the CNS diminished significantly, while jaw expression became visible. Subcloning of foxP2-enhancerA to yield enhancerA.1 and A.2 revealed very similar patterns to foxP2-enhancerA (Figure 5C–F). However, enhancerA.2 had significantly fewer labeled cells, suggesting that the more proximal region of enhancerA and enhancerA.1 contains necessary elements. foxP2-enhancerB expression in the telencephalon began at 24 hpf, with eye and hindbrain expression apparent by 72 hpf (Figure 5G, H). foxP2-enhancerD expression in the eye, dorsal diencephalon, and tectum began at 36 hpf, was maximal at 48 hpf, and decreased by 72 hpf (Figure 5I, J).

The enhancers we have identified partially mirror endogenous *foxP2 *expression [7]. While the enhancer fragments were chosen based on *in silico *prediction of potential *lef1 *binding sites [25], our analysis shows the importance of *in vivo *validation, since only 3 of the 6 predicted binding sites appear to lie in functional enhancer elements for the stages analyzed.

### *lef1 *knockdown leads to loss of expression from foxP2-enhancers A.1, B, and D

**Figure 6:**
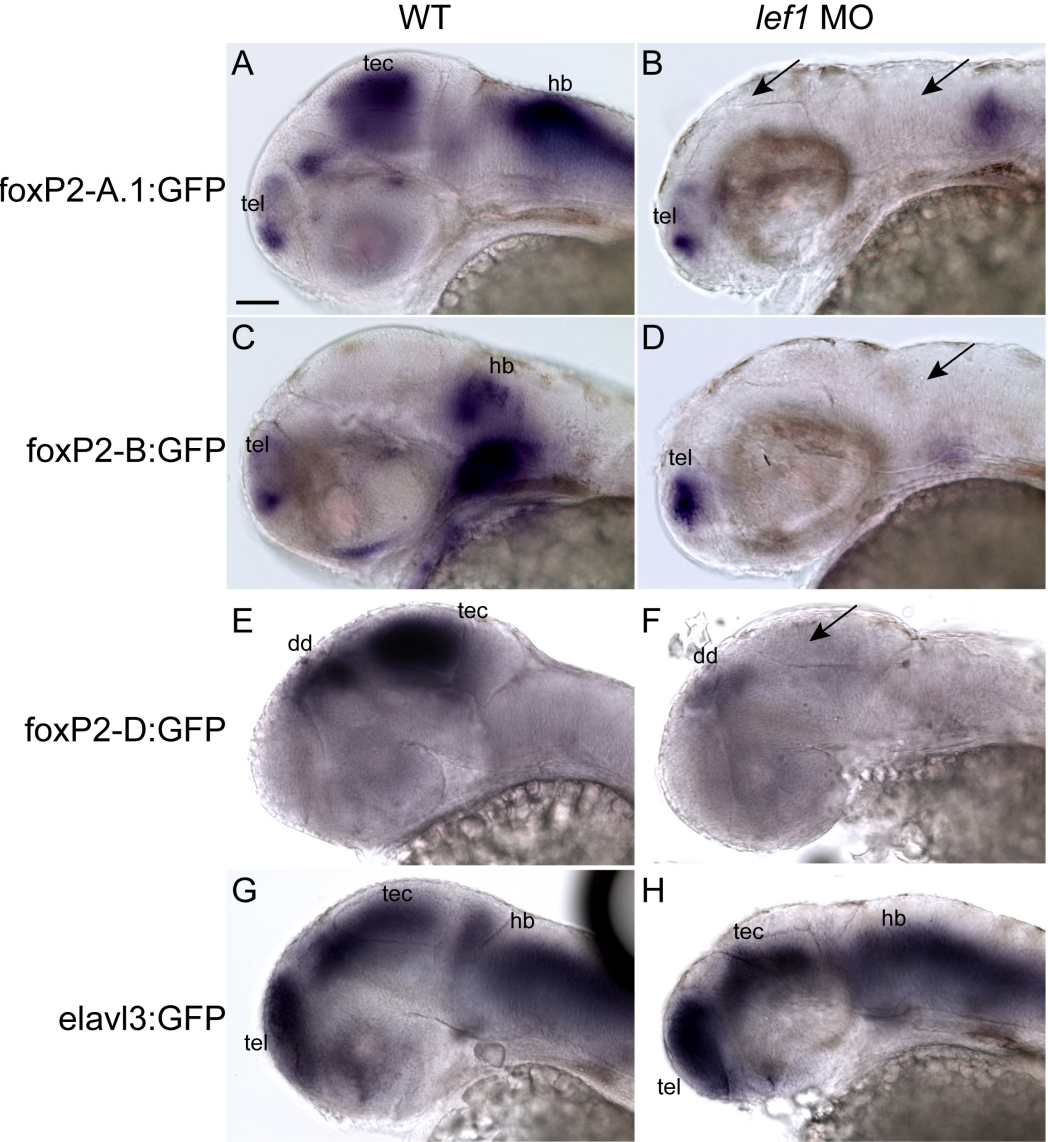
***lef1 *is necessary for expression from foxP2-enhancerA.1, foxP2-enhancerB, and foxP2-enhancerD**. Whole-mount *gfp in situ*s at 36 hpf; anterior to left, dorsal up, eyes removed. Scale bar = 50 μm. Conditions (wild type, wt; or *lef1 *morphant, *lef1 *MO) are shown above the panels, enhancer names to left. (Abbreviations: dd, dorsal diencephalon; hb, hindbrain; tec, tectum; tel, telencephalon.) (A) Tg*(foxP2-enhancerA.1:EGFP)*^*zc44 *^expresses in telencephalon, tectum, and hindbrain. (B) Tg*(foxP2-enhancerA.1:EGFP)*^*zc44 *^embryo injected with *lef1 *morpholino lacks GFP expression in tectum and hindbrain (*arrows*), although telencephalic expression persists. (C) Tg(*foxP2-enhancerB:EGFP*)^*zc41 *^expresses in telencephalon and hindbrain. (D) Tg(*foxP2-enhancerB:EGFP*)^*zc41 *^embryo injected with *lef1 *morpholino lacks GFP expression in the hindbrain (*arrow*), but telencephalic expression is present. (E) Tg*(foxP2-enhancerD:EGFP)*^*zc47 *^expresses in dorsal diencephalon and tectum. (F) Tg*(foxP2-enhancerD:EGFP)*^*zc47 *^embryo injected with *lef1 *morpholino lacks GFP expression in the tectum (arrow), but dorsal diencephalic expression persists. (G) Tg(*elavl3:EGFP*)^*zf8 *^embryo shows GFP expression in all post-mitotic neurons. (H) Tg(*elavl3:EGFP*)^*zf8 *^embryo injected with *lef1 *morpholino still has GFP expression in the tectum and hindbrain.

Based on our observations that foxP2-enhancers A.1, B, and D show expression in a pattern mirroring *lef1 *expression in the tectum and hindbrain, and contained putative binding sites for Lef1, we tested whether knockdown of *lef1 *led to loss of GFP expression. We injected *lef1 *morpholino into stable transgenic *foxP2 *enhancer lines (Tg*(foxP2-enhancerA.1:EGFP*)^zc44^, Tg*(foxP2-enhancerB:EGFP)*^*zc*41^, Tg*(foxP2-enhancerD:EGFP)*^*zc*47^), and looked for GFP expression in the tectum and hindbrain at 36 hpf. To detect enhancer-driven transcription with maximum sensitivity, we used an *in situ *probe for *gfp*. Morphants showed a loss of GFP expression in the tectum and hindbrain, but maintenance of telencephalic expression, in 89% of enhancerA.1 embryos (n = 71), 100% of enhancerB embryos (n = 22), and 100% of enhancerD embryos (n = 27) (Figure 6A–F). In contrast, in uninjected embryos only 2%, 0%, and 0% (n = 58, 15, and 16), respectively, showed loss of tectum and hindbrain expression. Further, the *lef1*-mediated loss of expression from the enhancers was visible at 52 hpf by visualization of GFP (Additional File 2).

To demonstrate that loss of GFP expression in the *lef1 *morphants was not simply due to absence of the GFP-expressing cells, we injected *lef1 *morpholino into transgenic fish lines Tg(*elavl3:EGFP*)^*zf8 *^(also known as *HuC:GFP*) and Tg*(pax2a:GFP*)^*e1*^. Tg(*elavl3:EGFP*)^*zf8 *^expresses GFP in all post-mitotic neurons [35], while Tg*(pax2a:GFP*)^*e1 *^expresses GFP in a pattern mirroring *pax2a *expression, including in the MHB, cerebellum, and hindbrain [36]. In both lines we found that *lef1 *morphants still expressed GFP in the tectum, MHB, and hindbrain (Figure 6G, H, and Additional File 2). In addition, using Tg(*isl3:GFP*)^zc7 ^to label retinal axons (A. Pittman and C.B.C., unpublished), we observed that the pattern of retinotectal projections appeared normal in 48 hpf morphants (data not shown). Although overall numbers of GFP-expressing cells appeared reduced in *lef1 *morphants of Tg(*elavl3:EGFP*)^*zf8 *^and Tg*(pax2a:GFP*)^*e1*^, in the *foxP2 *enhancer lines there was a complete lack of GFP-expressing cells in the tectum and hindbrain. These results show that loss of tectal and hindbrain expression observed with the *lef1 *morpholino in foxP2-enhancerA.1, foxP2-enhancerB, and foxP2-enhancerD is not simply due to a loss of neurons, but instead indicates a requirement for *lef1 *in these enhancers' function.

### Chromatin immunoprecipitation identifies critical Lef1 binding sites in foxP2-enhancerA.1 and foxP2-enhancerB

**Figure 7:**
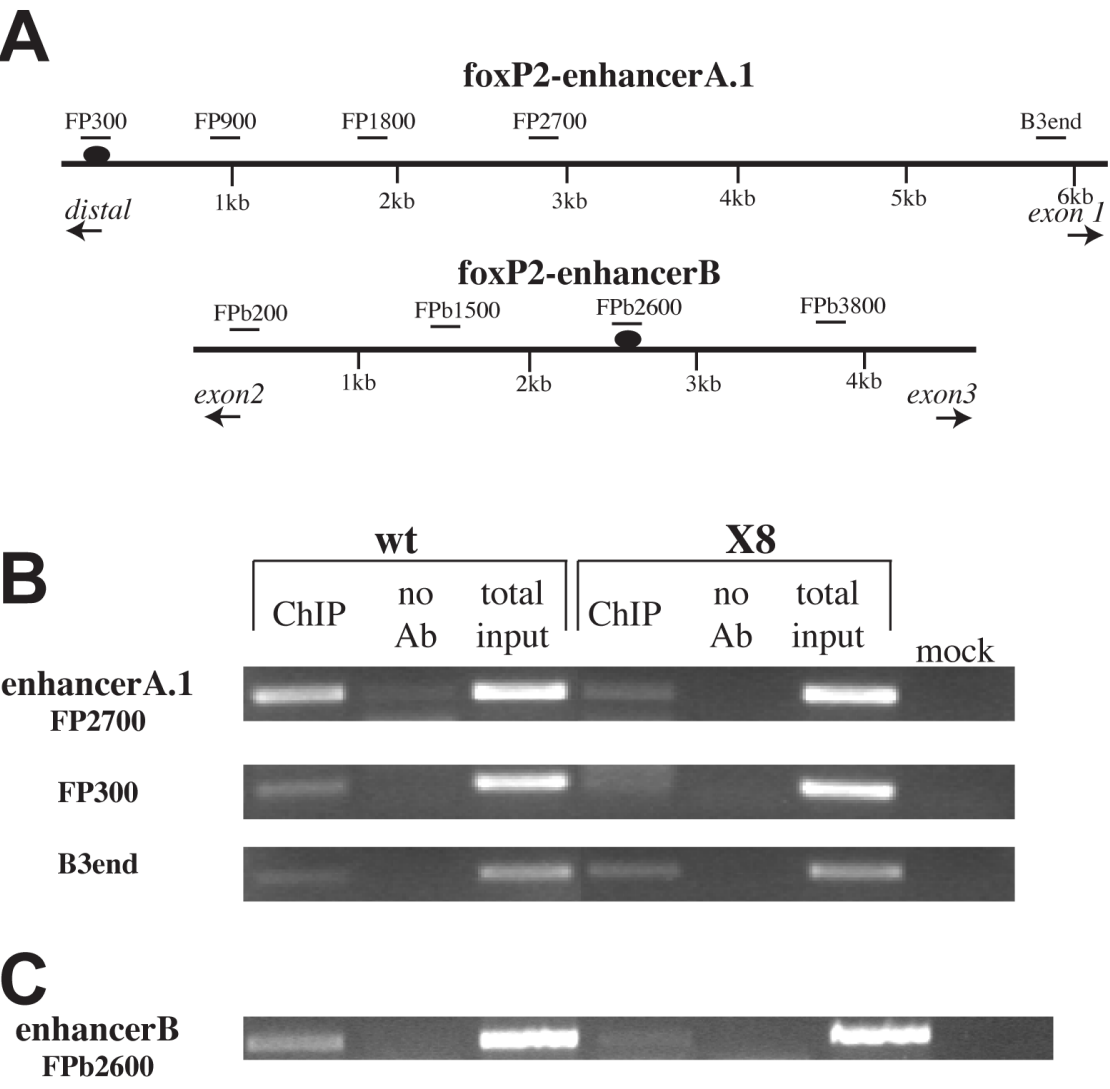
**ChIP analysis of foxP2-enhancerA.1 and foxP2-enhancerB genomic regions at 30 hpf**. (A) Diagram of foxP2-enhancerA.1 and foxP2-enhancerB regions. PCR amplicon locations and names are indicated above the genomic region; predicted Lef1 binding sites are shown as ovals. (B) Agarose gel analysis of ChIP PCR for foxP2-enhancerA.1, showing the PCR products for FP2700, FP300, and B3end. In wild type (wt) embryos, ChIP shows significant enrichment of the FP2700 product and slight enrichment of the FP300 product, compared to both the no antibody (Ab) control, and the ChIP of homozygous Df(*LG01*)^x8 ^embryos (Mock: water control for the PCR reaction.) In contrast, the B3end product showed no enrichment relative to the Df(*LG01*)^x8 ^embryos. (C) Agarose gel analysis of ChIP PCR for foxP2-enhancerB, showing the FPb2600 PCR product. In wt embryos, ChIP shows significant enrichment of the FPb2600 product, compared to both the no antibody and homozygous Df(*LG01*)^x8 ^controls.

To test whether Lef1 can bind to foxP2-enhancerA.1 and foxP2-enhancerB to regulate *foxP2 *expression, we performed chromatin immunoprecipitation (ChIP) using a polyclonal antibody against zebrafish Lef1 [27]. We designed PCR amplicons to test different regions of foxP2-enhancerA.1 and foxP2-enhancerB (Figure 7A), using chromatin extracted from 30 hpf embryos. For foxP2-enhancerA.1, we found that PCR amplicon FP2700 (centered around base pair 2700 in the enhancerA.1 DNA fragment) showed significant enrichment compared to controls (no antibody, and extracts from homozygous Df(*LG01*)^x8 ^embryos) (Figure 7B). Interestingly, amplicon FP300, encompassing the predicted Lef1 binding site in foxP2-enhancerA.1 (Figure 7A), showed minimal enrichment (Figure 7B). Further, a deletion construct of foxP2-enhancerA:GFP, removing the region containing FP300, did not cause loss of GFP expression (data not shown). This suggests that maximal Lef1 binding for foxP2-enhancerA.1 is centered around FP2700. For foxP2-enhancerB, we found that FPb2600 had significant enrichment compared to controls (Figure 7C). This PCR amplicon contains a predicted Lef1 binding site [25] (Figure 7A). We therefore conclude that Lef1 directly interacts with regulatory regions of *foxP2 *in 30 hpf zebrafish embryos.

## Discussion

Our data show that *lef1 *and *foxP2 *expression overlap in the developing CNS, and that *lef1 *is necessary for *foxP2 *expression in the tectum, MHB, and hindbrain. We identified Lef1binding sites in the *foxP2 *regulatory regions, based on the identification of *foxP2 *enhancers that depend on *lef1 *for expression, and ChIP analysis showing binding of Lef1 to these enhancers.

**Figure 8:**
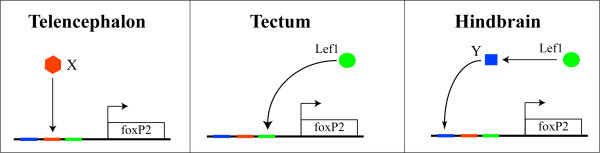
**Model of *foxP2 *regulation in the CNS**. In the telencephalon, an unknown factor X activates *foxP2 *expression. In the tectum (and MHB) Lef1 directly binds to enhancer elements of *foxP2*, leading to *foxP2 *expression. In the hindbrain expression of *foxP2 *is Lef1-dependent, but indirectly, since Lef1 is not expressed in the hindbrain.

*lef1 *regulation appears to be a combination of both direct and indirect effects on *foxP2 *(Figure 8). In the tectum and MHB, *lef1 *expression precedes *foxP2 *expression. Loss of *lef1 *leads to loss of both endogenous *foxP2 *expression, and expression from *foxP2 *enhancers. In the hindbrain loss of *lef1 *also leads to a loss of *foxP2 *expression. Since *lef1 *expression is not detectable in the hindbrain, we hypothesize that loss of *foxP2 *expression occurs both via an inductive effect of *lef1 *expression in the MHB, and through a second gene regulated by *lef1 *(Figure 8). This model would explain why we observe *foxP2 *expression (both by *in situ *and by expression from its enhancers) in the hindbrain (where *lef1 *is not expressed), as well as why *foxP2 *expression persists after *lef1 *expression in the MHB stops. In addition, our data demonstrate that *foxP2 *expression in the telencephalon, including the subpallium/basal ganglia, is regulated independently of *lef1*.

Since *foxP2 *expression in the telencephalon is not *lef1*-dependent, other factors must regulate *foxP2*. Conversely, in regions where *lef1 *is expressed but *foxP2 *is not, for example the dorsal diencephalon, lack of *foxP2 *expression could either reflect an absence of some necessary co-factors, or the presence of an inhibitor of expression, or both mechanisms. We have tried to globally activate *foxP2 *by heat-shock induction of a constitutively active form of *tcf3 *(RID, unpublished data), but did not observe any ectopic *foxP2 *expression (data not shown), implying stringent, multifactorial control of *foxP2 *expression.

Previous computer algorithm-based methods identified several other potential Lef1 binding sites in the *foxP2 *genomic region [25], but our *in vivo *analysis failed to support a role for them. Another recently described *in silico *method to identify transcription factor target genes would also fail to identify the *lef1 *enhancers for *foxP2*, as this method only included the 5 kb regions immediately upstream of transcriptional start sites [37]. Our work shows the importance of actual *in vivo *testing for analysis of enhancer gene regulation.

Since we analyzed expression up to 96 hours after fertilization, enhancers responsible for controlling expression at later times might have been missed. Given that all of the CNS domains of expression for *foxP2 *are present by 72 hpf [7,11], we feel that this is unlikely. However, we can not exclude the possibility that untested genomic regions might regulate expression, or that several independent regions might function in concert.

This study identified three regions functioning as enhancers for *foxP2*, specifically driving expression in the telencephalon, eye, diencephalon, tectum, and hindbrain. Their expression reveals that *foxP2 *is expressed in multiple brain regions, under strict temporal and spatial control, regulated through both *lef1*-dependent and -independent mechanisms. These transgenic lines label multiple distinct axon tracts (data not shown), and will allow analysis of subsets of *foxP2 *neurons and axons, in both wild type and mutant backgrounds, as well as misexpression and rescue experiments.

The role of *foxP2 *in CNS development has remained elusive, despite multiple genetic studies in humans demonstrating its necessity for normal language development [1-6]. In affected members of the human KE family, who carry a heterozygous point mutation which disrupts the function of one copy of *FOXP2*, voxel-based morphometric MRI analyses suggest disturbances in Broca's area, the basal ganglia, and the cerebellum [38,39]. Studies of *FoxP2 *knockout or point mutation mice have consistently shown cerebellar involvement. Homozygotes have been reported to have disordered Purkinje cell layering [17-19,40] and smaller cerebellar size [18,20], while heterozygotes are noted to display impaired motor learning and altered Purkinje cell synaptic plasticity [19,20]. Our identification of *lef1 *as a regulator of *foxP2 *expression in the MHB and hindbrain is a first step towards understanding the *foxP2 *genetic network involved in cerebellar development. The role of the cerebellum in language function is at least partially understood [41], but it is uncertain whether the oromotor apraxia of the KE family is due to defects of cerebellar or striatal pathways (or both) [40,42,43]. Importantly, other domains of *foxP2 *expression, for example in the telencephalon (including the subpallium), appear to be regulated independently of *lef1*.

## Conclusion

Wnt signaling has multiple roles in neuronal specification and the development of connectivity in the CNS [26], as does *lef1*, which is activated by the canonical Wnt signaling cascade. *lef1 *is necessary for development of dentate gyrus neurons [44], for neurogenesis and specification of neuronal subsets in the hypothalamus [27], and for expression of the transcription factors *zic2a *and *zic5 *in the tectum [45]. Here we show that *lef1 *regulates *foxP2 *expression in the tectum, MHB, and hindbrain. Interestingly, ChIP studies suggest that *FOXP2 *may in turn regulate components of the Wnt signaling pathway [23,24]. The identification of components of the *foxP2 *signaling cascade, including upstream, interacting, and downstream members, will be important for understanding *foxP2 *function, and for elucidating the genes and neural circuits involved in language development.

## Methods

### Fish stocks and embryo raising

Adult fish were bred according to standard methods. Embryos were raised at 28.5°C in E3 embryo medium with 0.003% phenylthiourea to inhibit pigment formation and staged by time and morphology [46]. For *in situ *staining, embryos were fixed in 4% paraformaldehyde (PFA) (in PBS) for 3 h at room temperature (RT) or overnight (O/N) at 4°C, washed briefly in PBS, dehydrated, and stored in 100% MeOH at -20°C until use.

Transgenic fish lines and alleles used were as follows: Df(*LG01*)^x8 ^[29]; Tg*(hsp70l:Δtcf-GFP)*^*w26 *^[30]; Tg*(foxP2-enhancerA:EGFP)*^*zc42*^; Tg*(foxP2-enhancerA.1:EGFP)*^*zc44*^; Tg(*foxP2-enhancerA.2:EGFP*)^*zc46*^; Tg*(foxP2-enhancerB:EGFP)*^*zc41*^; Tg*(foxP2-enhancerD:EGFP)*^*zc47*^; Tg*(pax2a:GFP*)^*e1 *^[36]; Tg(*elavl3:EGFP*)^*zf8 *^[35]. Df(*LG01*)^x8 ^homozygotes were identified by their smaller forebrain and flattened hindbrain phenotype (J.E.L. and R.I.D., unpublished). Tg*(hsp70l:Δtcf-GFP)*^*w26 *^embryos were identified by GFP expression after heat shock.

Heat shock was performed by incubation of 32 hpf embryos for 1 hour at 37°C, then collecting 3 hours after the end of the heat shock.

### *In situ *hybridization

Whole-mount *in situ *labeling for *foxP2*, *gfp*, and *lef1 *was performed as previously described [7,27,47]; the *mbx *RNA probe derived from full-length *mbx *cDNA cloned into pBluescript [31]. Double *in situs *were performed using a DNP-labeled probe for *foxP2 *and digoxigenin-labeled probe for *lef1*. Following standard hybridization and washes, DNP probe was detected using an anti-DNP HRP conjugate diluted 1:200 in TNTB block at 4°C overnight (PerkinElmer; [48]), followed by detection using Alexa Fluor 488 Tyramide diluted 1:250 (Molecular Probes) with 0.0015% hydrogen peroxide for 1 hour. Embryos were washed in TNT, blocked in TNTB, incubated with anti-digoxigenin alkaline phosphatase diluted 1:5000 in TNTB at 4°C overnight, and then detected with a standard BM Purple color reaction.

### Genomic PCR and Enhancer Cloning

PCR primers to clone *foxP2 *genomic fragments were as follows (forward and reverse primers, sequences 5' to 3'; size in kb listed immediately following the enhancer name): foxP2-enhancerA (9.7 kb): FP2.12L GTCGTAATTGCTCGGTGAC, FP2.3R GTGTGAATGCCAGCGATAGA; foxP2-enhancerA.1 (6.8 kb): FP2.12L, FP2.16R CGTCTCGACTGAGCAGAGTT; foxP2-enhancerA.2 (5.1 kb): FP2.12L, FP2.46R ACAACTGGCGTGTAAGGTGT; foxP2-fragment A.3 (4.7 kb): FP2.41L GACACCTTACACGCCAGTTG, FP2.3R; foxP2-fragment A.4 (2.3 kb): FP2.41L, FP2.40R CAGGGTGTGTTATAAACATGCAT; foxP2-enhancerB (4.6 kb): FP2.39L GACACTCTGGAGGAACTATG, FP2.38R GGAAACGGTGCAGTATGTGT; foxP2-fragment C (1.6 kb): F.FP1 GGCGGGTACCTGGTCATATT, F.RP2 TTTCCACCCAACCATAAATCA; foxP2-enhancerD (1.2 kb): H.FP1 CCAGCTATCCGAGAGGTTCA, H.RP2 CCGCCTGTTCAAATCAGAAT; foxP2-fragment E (0.69 kb): G.FP1 TGACCTCTGTGTAGCCTTGC, G.RP2 CATTGCTAGGGGAACGTGAT.

PCR was performed using standard conditions (TaKaRa LA PCR amplification kit 2.1, Millipore) from total genomic DNA, and PCR fragments gel purified prior to cloning (Qiagen gel purification kit). BP and LR reactions (Gateway cloning system, Invitrogen) were performed to clone the DNA fragments upstream of an adenovirus E1b minimal promoter and carp β-actin transcriptional start fused to EGFP (pENTRbasEgfp) in a Tol2 plasmid backbone (pTolR4-R2) [32,33,49]. BP reactions were performed by adding attB4 sequence to the 5' primer (5'-GGGGACAACTTTGTATAGAAAAGTTG-gene specific primer-3'), and attB1 sequence to the 3' primer (5'-GGGGACTGCTTTTTTGTACAAACTTG-gene specific primer-3'). The identity of the genomic fragments was confirmed by restriction enzyme digests and partial sequencing.

Injection of DNA constructs and raising of stable transgenic lines was performed essentially as described [33,49]. 20 pg of each enhancer:EGFP-Tol2 construct was co-injected with 20 pg of Tol2 transposase RNA in a total volume of 1 nL at the 1-cell stage. Embryos were screened for GFP expression from 12 hpf through 96 hpf. Patterns of enhancer expression were confirmed by multiple independent transient injections of the plasmid (> 5 injections for all constructs, > 100 embryos per injection), as well as isolation of 2 or more independent stable transgenic lines (in cases where stable transgenics were isolated).

### Morpholino oligonucleotide sequences

The *lef1 *and *tcf3b *splice-blocking morpholinos (MO) were synthesized by Gene Tools (Philomath, OR); efficacy and use were as previously described [27,28,50,51]. For *lef1*, 2 ng of morpholino E7I7 was injected; for *tcf3b*, 5 ng was injected.

### Microscopy and image analysis

Whole-mount images were taken using brightfield microscopy with embryos mounted in 80% glycerol. Confocal microscope images of live embryos were taken after mounting embryos anesthetized using tricaine (0.004%) in 1.5% low melt agarose dissolved in 0.33× PBS/7.3% glycerol. Image acquisition and analysis were performed as described previously [52]. Confocal images of double *in situ*s were taken on an Olympus FV1000 after mounting embryos in 80% glycerol. BM Purple fluorescence was imaged with 633 nm excitation, collecting emission from 700–800 nm [53].

### Sequence Analysis

We used coordinate conversion ("convert" function) in the UCSC genome web server [54] to identify conserved *lef1 *binding sites in the *foxP2 *region of zebrafish, based on predicted Tcf4 binding sites positions in the human genome (since Tcf4 and Lef1 bind to a shared motif) [25]. The genomic upstream sequence of *foxP2 *was obtained by performing a BLAST search against unfinished sequences in the Sanger Centre *Danio rerio *sequencing project (sequence for foxP2-enhancerA is contained in BAC DKEY-116L11; GenBank accession CU468733). To identify the sixth potential Lef1 binding site in the foxP2-enhancerA region predicted by Hallikas et al. [25], we used the enhancer element locator algorithm (available at , and compared the 9 kb regions upstream of the first coding exon of human and zebrafish *FOXP2 *using the Tcf4 consensus binding motif.

### Chromatin immunoprecipitation (ChIP)

ChIP was performed as previously described [27,55] with the following modifications. 80–120 embryos of each genotype (wild type or homozygous Df(*LG01*)^x8^) were collected between 28 hpf and 30 hpf, dechorionated, and fixed in 2.2% formaldehyde for 15 minutes at room temperature. Embryos were rinsed in 0.125 M glycine, followed by PBS, and then lysed. The Df(*LG01*)^x8 ^lysate was checked by genomic PCR for the *lef1 *gene to confirm that no PCR product was obtained. PCR products were visualized on ethidium bromide-stained agarose gels. Primers used for ChIP analysis of the foxP2-enhancerA.1 genomic region were the following (forward and reverse primers, sequences 5' to 3'): FP300, CGACTCTCCGCGAAACAC, CATTGCCATTATCACCAGCA; FP900, CCTATCAAAGGCAGGCAGA, TTATCGCACTAAACAAGCTATTACAC; FP1800, CACAGAGTGAAGTCATGGAGAAA, AGCGCACGCACAACTAATC; FP2700, AGAGAAACGGATAAACAGTGAGAAG, CCCTCTCGAACCCTCAAAA; B3end, TGATTAACGCCGTCTTTTCC, AGCGCACGCACAATAAAATA. Primers used for ChIP analysis of the foxP2-enhancerB genomic region were (forward and reverse primers, sequences 5' to 3'): FPb200, AGCTGGAAGGAAGTGTCTGG, TTTTGGCATGTGCAAAGAAG; FPb1500, GCGTATGTATGCTTGTCAGGTT, TGCGTGGTGTTTTACTTGGA; FPb2600, CAGATCGACGGATGATACACA, TTCCGCCAATTATCATGTCA; FPb3800, GACCCCTTCGCAATGTCTAAT, TTCGAGAAATGCTTGCACAC.

## Abbreviations

ChIP: chromatin immunoprecipitation; CNS: central nervous system; MHB: mid-hindbrain boundary;

## Competing interests

The authors declare that they have no competing interests.

## Authors' contributions

JLB and EF jointly carried out cloning and *in situ*s. XW performed ChIP. JLB and JEL performed expression analysis. JLB, CBC, and RID conceived the study, and participated in its design and coordination. JLB was largely responsible for writing the manuscript. All authors read and approved the final manuscript.

## Supplementary Material

Additional file 1 **Loss of *lef1 *leads to absent *foxP2 *expression in the tectum, mid-hindbrain boundary, and hindbrain**. Whole-mount *in situ*s at 48 hpf; anterior to left, dorsal up, eyes removed. Scale bar = 50 μm. (Conditions: hs:Δtcf- Tg*(hsp70l:Δtcf:GFP*)^w26^, *lef1 *mo- *lef1 *morphants, wt- wild type, x8- homozygous Df(*LG01*)^x8^. Abbreviations: hb: hindbrain; mhb: mid-hindbrain boundary; tec: tectum; tel: telencephalon.) (A, B) *lef1 *morphant (B) lacks expression in tectum and has diminished expression in the hindbrain (*arrows*), compared to wild-type (A). (C) In Df(*LG01*)^x8 ^homozygote, expression is absent in the tectum and hindbrain (*arrows*). (D) In Tg*(hsp70l:Δtcf:GFP*)^w26 ^embryo, collected four hours after heat shocking, expression is absent from the tectum and hindbrain (*arrows*). MHB expression is reduced but not absent, perhaps because *lef1 *induction of *foxP2 *expression is already sufficiently established by the time of heat-shock. Click here for file

Additional file 2 ***lef1 *is necessary for expression from foxP2-enhancerA.1, foxP2-enhancerB, and foxP2-enhancerD**. Whole-mount confocal images at 52 hpf (except foxP2-enhancerD), lateral view, anterior left, dorsal up. Conditions (wild type- wt, or *lef1 *morphant- *lef1 *MO) are shown above the panels, while the enhancer name is to the left of the panels. (Abbreviations: hb: hindbrain; tec: tectum; tel: telencephalon.) (A) Tg*(foxP2-enhancerA.1:EGFP)*^*zc44 *^expresses in telencephalon, tectum, and hindbrain. (B) Tg*(foxP2-enhancerA.1:EGFP)*^*zc44 *^embryo injected with *lef1 *morpholino: GFP expression is absent in the tectum and hindbrain (*arrows*), although telencephalic expression persists. (C) Tg(*foxP2-enhancerB:EGFP*)^*zc41 *^expresses in telencephalon, tectum, and hindbrain. (D) Tg(*foxP2-enhancerB:EGFP*)^*zc41 *^embryo injected with *lef1 *morpholino: GFP expression is absent in the hindbrain (*arrow*), but still present in the telencephalon. (E) Tg*(foxP2-enhancerD:EGFP)*^*zc47 *^expresses in tectum. (F) Tg*(foxP2-enhancerD:EGFP)*^*zc47 *^embryo injected with *lef1 *morpholino: GFP expression is absent in the tectum (arrow). (G) Tg(*elavl3:EGFP*)^*zf8 *^embryo shows GFP expression in all post-mitotic neurons. (H) Tg(*elavl3:EGFP*)^*zf8 *^embryo injected with *lef1 *morpholino: GFP expression is still present in the tectum and hindbrain. (I) Tg*(pax2a:GFP*)^*e1 *^embryo shows expression in the MHB and hindbrain. (J) Tg*(pax2a:GFP*)^*e1 *^embryo injected with *lef1 *morpholino has persistent expression in the MHB and hindbrain. Click here for file
