# Supplementary figures and images for: Domain-specific regulation of foxP2 CNS expression by lef1
Source: BMC Dev Biol. 2008 Oct 24;8:103. doi: 10.1186/1471-213X-8-103 (PMC2579431; doi:10.1186/1471-213X-8-103)

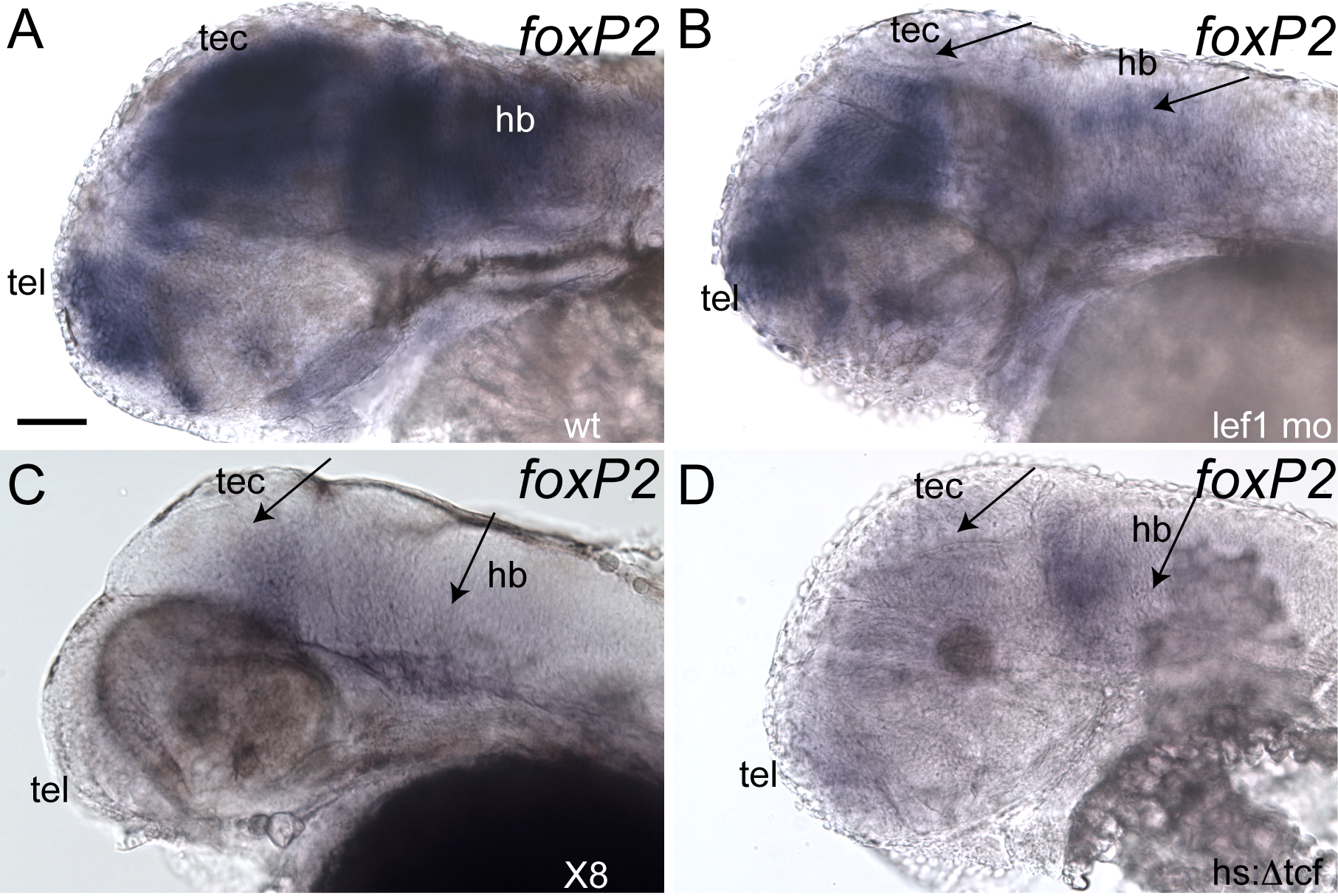

Supplement: Additional file 1 — Loss of lef1 leads to absent foxP2 expression in the tectum, mid-hindbrain boundary, and hindbrain. Whole-mount in situs at 48 hpf; anterior to left, dorsal up, eyes removed. Scale bar = 50 μm. (Conditions: hs:Δtcf- Tg(hsp70l:Δtcf:GFP)w26, lef1 mo- lef1 morphants, wt- wild type, x8- homozygous Df(LG01)x8. Abbreviations: hb: hindbrain; mhb: mid-hindbrain boundary; tec: tectum; tel: telencephalon.) (A, B) lef1 morphant (B) lacks expression in tectum and has diminished expression in the hindbrain (arrows), compared to wild-type (A). (C) In Df(LG01)x8 homozygote, expression is absent in the tectum and hindbrain (arrows). (D) In Tg(hsp70l:Δtcf:GFP)w26 embryo, collected four hours after heat shocking, expression is absent from the tectum and hindbrain (arrows). MHB expression is reduced but not absent, perhaps because lef1 induction of foxP2 expression is already sufficiently established by the time of heat-shock. [file 1471-213X-8-103-S1.tiff]

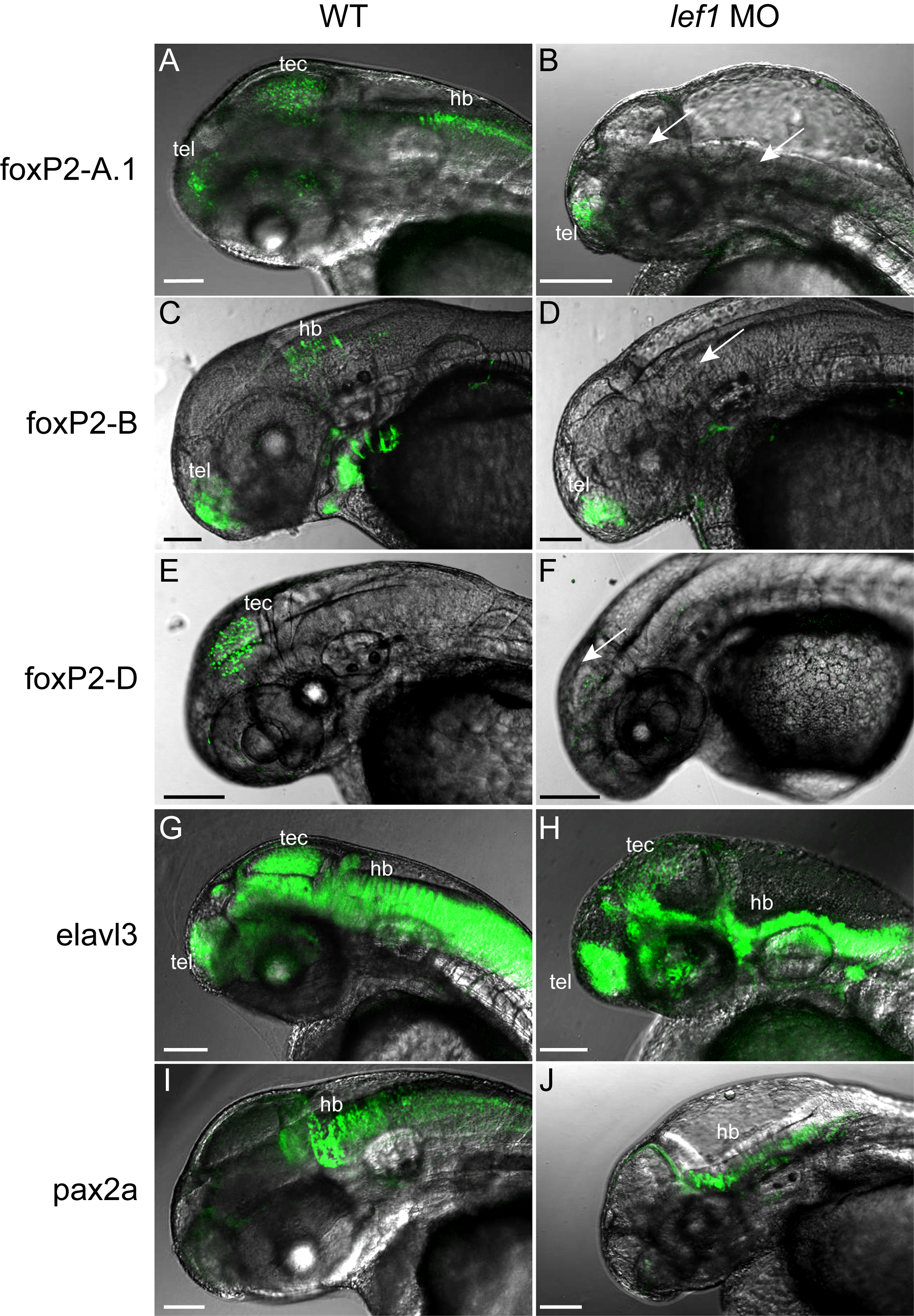

Supplement: Additional file 2 — lef1 is necessary for expression from foxP2-enhancerA.1, foxP2-enhancerB, and foxP2-enhancerD. Whole-mount confocal images at 52 hpf (except foxP2-enhancerD), lateral view, anterior left, dorsal up. Conditions (wild type- wt, or lef1 morphant- lef1 MO) are shown above the panels, while the enhancer name is to the left of the panels. (Abbreviations: hb: hindbrain; tec: tectum; tel: telencephalon.) (A) Tg(foxP2-enhancerA.1:EGFP)zc44 expresses in telencephalon, tectum, and hindbrain. (B) Tg(foxP2-enhancerA.1:EGFP)zc44 embryo injected with lef1 morpholino: GFP expression is absent in the tectum and hindbrain (arrows), although telencephalic expression persists. (C) Tg(foxP2-enhancerB:EGFP)zc41 expresses in telencephalon, tectum, and hindbrain. (D) Tg(foxP2-enhancerB:EGFP)zc41 embryo injected with lef1 morpholino: GFP expression is absent in the hindbrain (arrow), but still present in the telencephalon. (E) Tg(foxP2-enhancerD:EGFP)zc47 expresses in tectum. (F) Tg(foxP2-enhancerD:EGFP)zc47 embryo injected with lef1 morpholino: GFP expression is absent in the tectum (arrow). (G) Tg(elavl3:EGFP)zf8 embryo shows GFP expression in all post-mitotic neurons. (H) Tg(elavl3:EGFP)zf8 embryo injected with lef1 morpholino: GFP expression is still present in the tectum and hindbrain. (I) Tg(pax2a:GFP)e1 embryo shows expression in the MHB and hindbrain. (J) Tg(pax2a:GFP)e1 embryo injected with lef1 morpholino has persistent expression in the MHB and hindbrain. [file 1471-213X-8-103-S2.tiff]
